# Cadonilimab rechallenge in patients with recurrent or metastatic cervical cancer following prior PD-1/PD-L1 inhibitor failure: a retrospective multicenter study

**DOI:** 10.3389/fimmu.2025.1701319

**Published:** 2026-01-09

**Authors:** Haijuan Yu, Jian Chen, Jie Lin, Lijun Chen, Jianping Zou, Bin Liu, Linying Liu, Ning Xie, Sufang Deng, Shengtao Zhou, Yang Sun

**Affiliations:** 1 Department of Gynecology, Clinical Oncology School of Fujian Medical University, Fujian Cancer Hospital, Fuzhou, Fujian, China; 2 Department of Obstetrics and Gynecology, Key Laboratory of Birth Defects and Related Diseases of Women and Children of Ministry of Education, West China Second Hospital, Chengdu, China

**Keywords:** cadonilimab, recurrent or metastatic cervical cancer, immunotherapy rechallenge, efficacy analyses, adverse events

## Abstract

**Purpose:**

Patients with recurrent or metastatic cervical cancer (R/M CC) who progress after immunotherapy face limited treatment options. This study aimed to explore whether cadonilimab, a novel bispecific antibody targeting programmed cell death protein 1 (PD-1)/cytotoxic T-lymphocyte antigen-4 (CTLA-4), could effectively treat such patients following PD-1/programmed death-ligand 1 (PD-L1) inhibitor failure.

**Methods:**

A retrospective multicenter study was conducted on 29 patients with R/M CC who received cadonilimab treatment after immune checkpoint inhibitor (ICI) failure between August 2022 and April 2024. The study assessed the objective response rate (ORR), the disease control rate (DCR), the progression-free survival (PFS), the overall survival (OS), and the safety profiles. Given the small sample size and its retrospective nature, this study is fundamentally descriptive, and its findings should be interpreted as exploratory.

**Results:**

Among the 29 patients, the ORR was 24.1% (7/29) and the DCR was 55.2% (16/29). The median PFS was 5.8 months, while the median OS was 12.1 months. Subgroup analyses identified poorer prognoses for patients with liver metastasis, those with three or more prior treatment lines, and those receiving cadonilimab monotherapy. The most common grade 3 or higher adverse events (AEs) were anemia [8 (27.6%)], decreased white blood cell count [4 (13.8%)], and decreased neutrophil count [4 (13.8%)].

**Conclusion:**

Cadonilimab might offer a promising option with a manageable safety profile for patients with R/M CC who progress after ICI treatment. Further studies with larger sample sizes are needed to confirm these findings.

## Introduction

Cervical cancer (CC) persists as a major global health burden, ranking fourth in both incidence and cancer-related mortality among women, with 661,021 new cases and 348,189 deaths reported in 2022 (1, 2). A major challenge in the treatment of CC lies in its high recurrence rate (15%–20%) (3) and primary metastatic presentation (6%) (4), leading to a detrimental prognosis even with high-intensity treatment (5). The advent of immune checkpoint inhibitors (ICIs) has revolutionized the management of patients with recurrent/metastatic (R/M) CC (6–8), based on the outstanding outcomes from the phase III, double-blind KEYNOTE-826 trial. Data showed that survival benefits could be substantially achieved with the addition of pembrolizumab regardless of bevacizumab [median overall survival (mOS)_bevacizumab-treated_ = 37.6 months *vs*. mOS_bevacizumab-naïve_ = 17.1 months] in patients with R/M CC (9). This marks the beginning of a new era in which more promising and effective immunotherapy drugs with fewer adverse events (AEs) are emerging, as seen in clinical trials such as the CheckMate 358 Trial (10), EMPOWER-Cervical1/GOG-3016/ENGOT-cx9 (11), and NCT04150575 (12).

Despite these advances, a significant proportion of patients still experience disease progression after the initial ICI treatment, highlighting the need for strategies to overcome immune resistance (13). In response, the concept of ICI rechallenge has emerged and has been applied in practice due to its outstanding efficacy in melanomas and lung cancers (14). ICI rechallenge refers to the reuse of ICIs after serious AEs or disease progression during/after a previous ICI treatment, including either single-agent or dual-agent immunotherapy. A deeper understanding of the resistance mechanisms of ICIs—including T-cell exhaustion, immune escape, and dynamic alterations within the tumor microenvironment (TME)—is essential for optimizing rechallenge strategies. Reintroducing a combination of other ICIs may help reverse T-cell exhaustion, reactivate antitumor immunity, and restore tumor sensitivity to treatment (14).

Cadonilimab, a novel bispecific antibody that targets programmed cell death protein 1 (PD-1) and cytotoxic T-lymphocyte-associated antigen 4 (CTLA-4) (15), has shown promising potential in overcoming immune resistance (16–18). Mechanistically, CTLA-4 inhibition promotes T-cell priming and expansion within the lymph nodes, while PD-1 blockade predominantly reverses T-cell exhaustion in the TME, resulting in a synergistic immune activation. This dual checkpoint inhibition enhances the immune activation even in tumors that previously failed to respond to PD-1 monotherapy (19). Moreover, a number of retrospective studies and case reports have also confirmed the promising role of cadonilimab in immunotherapy rechallenge (20, 21). Wang et al. (22) documented an ICI-pretreated lung cancer case who still achieved stable disease (SD) for 6 months with cadonilimab rechallenge. These findings suggest that cadonilimab may represent a promising rechallenge option for CC. However, clinical evidence remains insufficient with regard to the role of cadonilimab in previously ICI-treated R/M CC patients.

To address this gap, this study aimed to evaluate the therapeutic outcomes and safety profile of cadonilimab in patients with R/M CC who experienced disease progression or intolerance with ICI treatment. The findings could provide preliminary, hypothesis-generating evidence for clinical decision-making when dealing with this particular population.

## Methods

### Study design and participant selection

This multicenter retrospective cohort study analyzed 29 patients with R/M CC who received cadonilimab after prior immunotherapy failure across 10 tertiary medical institutions in China between August 2022 and April 2024, representing all consecutive eligible patients treated during the study period. The eligibility criteria were as follows: 1) age ≥18 years; 2) with histologically confirmed R/M CC, including squamous cell carcinoma, adenocarcinoma, adenosquamous carcinoma, or neuroendocrine carcinoma; 3) with documented disease progression or intolerance to prior ICI monotherapy; and 4) had at least one cycle of cadonilimab—concurrent therapies were permitted (e.g., radiotherapy, chemotherapy, or anti-angiogenic agents). Patients were excluded if their medical records during cadonilimab treatment were incomplete.

In this study, ICI rechallenge was defined as the initiation of cadonilimab following discontinuation of a prior ICI regimen, with an interval of at least 3 weeks from the last ICI dose. Prior ICI cessation was classified into three categories: 1) confirmed disease progression; 2) treatment interruption due to immune-related AEs; or 3) elective discontinuation after achieving clinical benefit.

### Ethical considerations

This study was conducted in accordance with the Declaration of Helsinki and Good Clinical Practices Guidelines. The protocol was approved by the Ethics Committee of Fujian Cancer Hospital (K2023-102-01) and was subsequently acknowledged or approved by the institutional review boards of all participating centers. Written informed consent was obtained from all patients. All patient data were anonymized and processed in compliance with applicable ethical and regulatory requirements.

### Treatment procedures

A unified electronic case report form was designed prior to data extraction. Treatment details, including the therapeutic regimens, the dose modifications, the assessment schedules, and the reasons for treatment discontinuation, were retrospectively collected from electronic medical records and from direct communication with the attending physicians. Cadonilimab was administered intravenously at a dose of either 10 mg/kg every 3 weeks or 6 mg/kg every 2 weeks, according to the physician’s discretion. Several patients with an Eastern Cooperative Oncology Group Performance Status (ECOG PS) of 3 were not excluded from cadonilimab therapy following clinical evaluation and shared clinical decision-making, as their PS decline was considered reversible and related to tumor burden. These patients were closely monitored during treatment initiation. Treatment was continued until radiologic evidence of disease progression, the occurrence of unacceptable toxicity, or discontinuation based on the decision of the treating physician or the patient (23).

### Efficacy evaluation

Radiologic tumor evaluation was performed using CT or MRI, with all participating centers applying the Response Evaluation Criteria in Solid Tumors (RECIST) version 1.1. Tumor response was reviewed by investigators and radiologists and classified into complete response (CR), partial response (PR), SD, or progressive disease (PD) (24). The primary endpoints included objective response rate (ORR), disease control rate (DCR), progression-free survival (PFS), overall survival (OS), and safety outcomes. ORR was defined as the proportion of patients who achieved CR or PR, whereas DCR represented the proportion who achieved CR, PR, or SD. PFS was measured from the first administration of cadonilimab to radiologically confirmed disease progression or death from any cause. OS was calculated from treatment initiation to death from any cause, loss to follow-up, or the last follow-up, whichever occurred first. Patients without documented progression or death at the data cutoff—including those lost to follow-up—were censored at the date of their last recorded clinical visit or imaging assessment. The data cutoff for survival analyses was April 30, 2024.

### Adverse event assessment

AEs were collected from electronic medical records and were categorized according to the Common Terminology Criteria for Adverse Events (CTCAE) version 5.0 (25). Events with a clinical pattern consistent with immune-mediated toxicity were classified as immune-related adverse events (irAEs). The management of AEs followed contemporary clinical practice guidelines. IrAEs were treated with dose interruption, corticosteroids, and additional immunosuppressive therapy, when indicated. Non-immune toxicities—including hematologic, gastrointestinal, and vascular AEs—were managed according to the causative agent, with dose adjustment or discontinuation of the relevant therapy at the discretion of the treating physician. Severe AEs such as myocarditis were managed with immediate treatment cessation, high-dose corticosteroids, and multidisciplinary consultation (26).

### Statistical analysis

Descriptive statistics presented the categorical variables as frequencies (percentage) and the continuous variables as the median (range). Survival curves were estimated using the Kaplan–Meier method with Greenwood’s formula and log-rank tests for between-group comparisons. The statistical significance threshold was set at two-tailed *p* < 0.05. Analyses were performed using SPSS 26.0, R software (version 4.3.1), and GraphPad Prism 6.0.1.

## Results

### Characteristics of the study population

**Figure 1:**
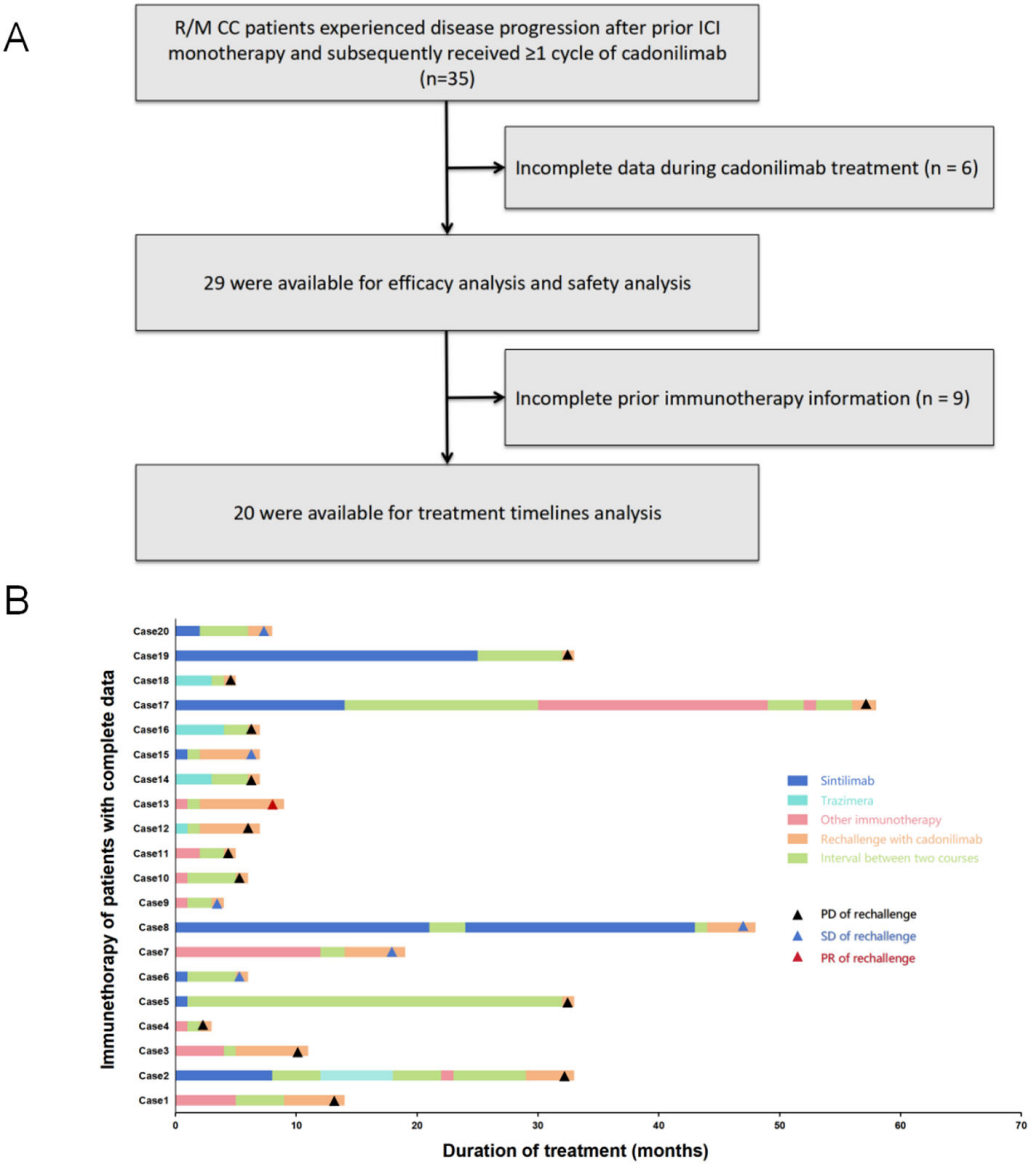
**(A)** Flow diagram of the patient selection. **(B)** Swimming plots of the patients with complete medical data. *PD*, progressive disease; *SD*, stable disease; *PR*, partial response.

From August 2022 to April 2024 (a period preceding the inclusion of cadonilimab in the 2024 National Reimbursement Drug List in China), 29 patients with R/M CC who had progressed after prior ICI monotherapy were retrospectively enrolled for efficacy and safety analyses. Of these, 20 patients were included in the treatment timeline analysis. The median time from the initial cancer diagnosis to the study enrollment was 36 months (range = 2–79 months) (**Figures 1A, B**).

The detailed baseline characteristics are summarized in **Table 1**. The median age was 53 years (range = 26–72 years). Majority of the patients had advanced disease, with 86.2% (25/29) classified as stage III or IV. The most common sites of metastatic involvement were lymph nodes (19/29, 65.5%), lungs (12/29, 41.4%), and bones (11/29, 37.9%). In addition, 51.7% (15/29) had received more than three prior lines of systemic therapy. Majority of the patients had previously been treated with PD-1 inhibitors and discontinued immunotherapy due to disease progression.

**Table 1:** Baseline characteristics of the 29 patients.

Characteristics	Patients (*N* = 29)
Age (years)	
Median (range)	53 (26–72)
Ethnicity, *n* (%)	
Chinese	29 (100)
ECOG performance status score, *n* (%)	
<2	15 (51.7)
≥2	14 (48.3)
FIGO stage at initial diagnosis, *n* (%)	
IIA	1 (3.4)
IIB	3 (10.3)
IIIA	0 (0.0)
IIIB	2 (6.9)
IIIC	10 (34.5)
IVA	2 (6.9)
IVB	11 (37.9)
Histopathological type, *n* (%)	
Squamous cell carcinoma	21 (72.4)
Adenocarcinoma	6 (20.7)
Adenosquamous carcinoma	1 (3.4)
Neuroendocrine carcinoma	1 (3.4)
PD-L1 CPS, *n* (%)	
Positive (CPS ≥ 1)	4 (13.8)
Negative (CPS < 1)	9 (31.0)
Unknown	16 (55.2)
Location of metastases, *n* (%)	
Local recurrence only	2 (6.9)
Distant metastasis only	22 (75.9)
Combined	5 (17.2)
Site of metastases, *n* (%)	
Lymph nodes	19 (65.6)
Pelvis	9 (31.0)
Lung	12 (41.4)
Bone	11 (37.9)
Liver	8 (27.6)
Previous lines of therapy, *n* (%)	
1–2	14 (48.3)
≥ 3	15 (51.7)
Previous radiotherapy, *n* (%)	
Yes	25 (86.2)
No	4 (13.8)
Previous systemic therapies, *n* (%)	
ICIs	29 (100)
AT	15 (51.7)
Paclitaxel	24 (82.8)
Platinum	26 (89.7)
Previous immunotherapy, *n* (%)	
PD-1 inhibitor	22 (75.9)
PD-1 inhibitor + PD-L1 inhibitor	2 (6.9)
Unknown	5 (17.2)
Reason for prior ICI cessation, *n* (%)	
Disease progression	27 (93.1)
Elective discontinuation after clinical benefit	1 (3.4)
Immune-related adverse events	1 (3.4)
Current systemic therapies, *n* (%)	
Cadonilimab only	9 (31.0)
Cadonilimab + CT	2 (6.9)
Cadonilimab + RT + CT + AT	3 (10.3)
Cadonilimab + CT + AT	5 (17.2)
Cadonilimab + AT	10 (34.5)

*ECOG*, Eastern Cooperative Oncology Group; *FIGO*, International Federation of Gynecology and Obstetrics; *PD-1*, programmed cell death protein 1; *PD-L1*, programmed death-ligand 1; *CPS*, combined positive score; *ICIs*, immune checkpoint inhibitors; *AT*, anti-angiogenic therapy; *CT*, chemotherapy; *RT*, radiotherapy.

During cadonilimab rechallenge, combination therapy was administered to 69.0% (20/29) of the patients. Among those receiving chemo-immunotherapy, the regimens included albumin-bound paclitaxel plus carboplatin (*n* = 4) or cisplatin (*n* = 1), paclitaxel plus cisplatin (*n* = 3), docetaxel plus nedaplatin (*n* = 1), and albumin-bound paclitaxel monotherapy (*n* = 1). Furthermore, 18 patients received anti-angiogenic therapy, including bevacizumab (*n* = 14) or anlotinib (*n* = 4). Three patients also underwent radiotherapy targeting either the primary tumor or the metastatic lesions.

### Efficacy analyses

**Figure 2:**
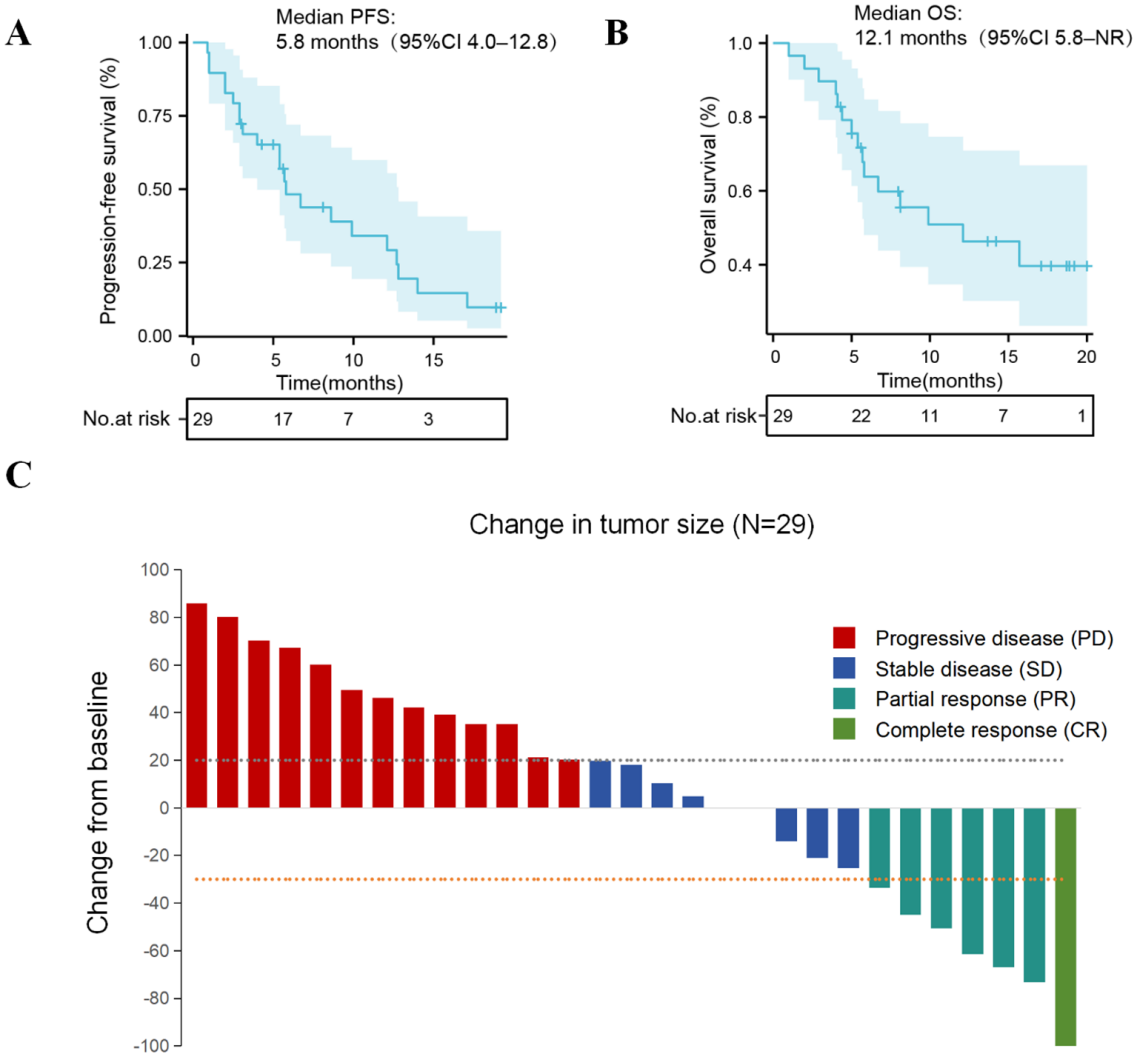
**(A)** Kaplan–Meier curves of the progression-free survival (PFS) for the entire group. **(B)** Kaplan–Meier curves of the overall survival (OS) for the entire group. **(C)** Tumor responses with cadonilimab rechallenge. *NR*, not reached.

Up to April 30, 2024, the median follow-up time was 16 months (range = 5–23 months). In this exploratory cohort, rechallenge with cadonilimab demonstrated signals of clinical activity, with a median PFS of 5.8 months (95%CI = 4.0–12.8) and a median OS of 12.1 months (95%CI = 5.8–not reached) (**Figures 2A, B**). Overall, one patient achieved CR and six achieved PR, resulting in an ORR of 24.1% (7/29) and a DCR of 55.2% (16/29) (**Figure 2C**). Notably, tumor response was observed in a small subset of programmed death-ligand 1 (PD-L1)-negative patients (75%, three of four cases) (**Table 2**).

**Table 2:** Response rates with cadonilimab rechallenge.

Response	Patients, *n* (%)					
	All (*N* = 29)	CPS ≥ 1 (*n* = 9)	CPS < 1 (*n* = 4)	Cadonilimab monotherapy (*n* = 9)	Combination with CT (*n* = 10)	Combination with AT (*n* = 18)
CR	1 (3.4)	1 (11.1)	0 (0)	0 (0)	1 (10.0)	1 (5.6)
PR	6 (20.7)	3 (33.3)	1 (25.0)	1 (11.1)	1 (10.0)	5 (27.8)
SD	9 (31.0)	2 (22.2)	2 (50.0)	3 (33.3)	4 (40.0)	6 (33.3)
PD	13 (44.8)	3 (33.3)	1 (25.0)	5 (55.6)	4 (40.0)	6 (33.3)
ORR	7 (24.1)	4 (44.4)	1 (25.0)	1 (11.1)	2 (20.0)	6 (33.3)
DCR	16 (55.2)	6 (66.7)	3 (75.0)	4 (44.4)	6 (60.0)	12 (66.7)

*CR*, complete response; *PR*, partial response; *SD*, stable disease; *PD*, progressive disease; *ORR*, objective response rate; *DCR*, disease control rate; *CT*, chemotherapy; *AT*, anti-angiogenic therapy.

To account for potential confounding from concomitant treatments, the response outcomes were further examined according to treatment modality. Among patients receiving cadonilimab monotherapy, the ORR was 11.1% (1/9). In comparison, the ORRs were 20.0% (2/10) in those who received cadonilimab with chemotherapy and 33.3% (6/18) in those treated with cadonilimab plus anti-angiogenic therapy (**Table 2**).

**Figure 3:**
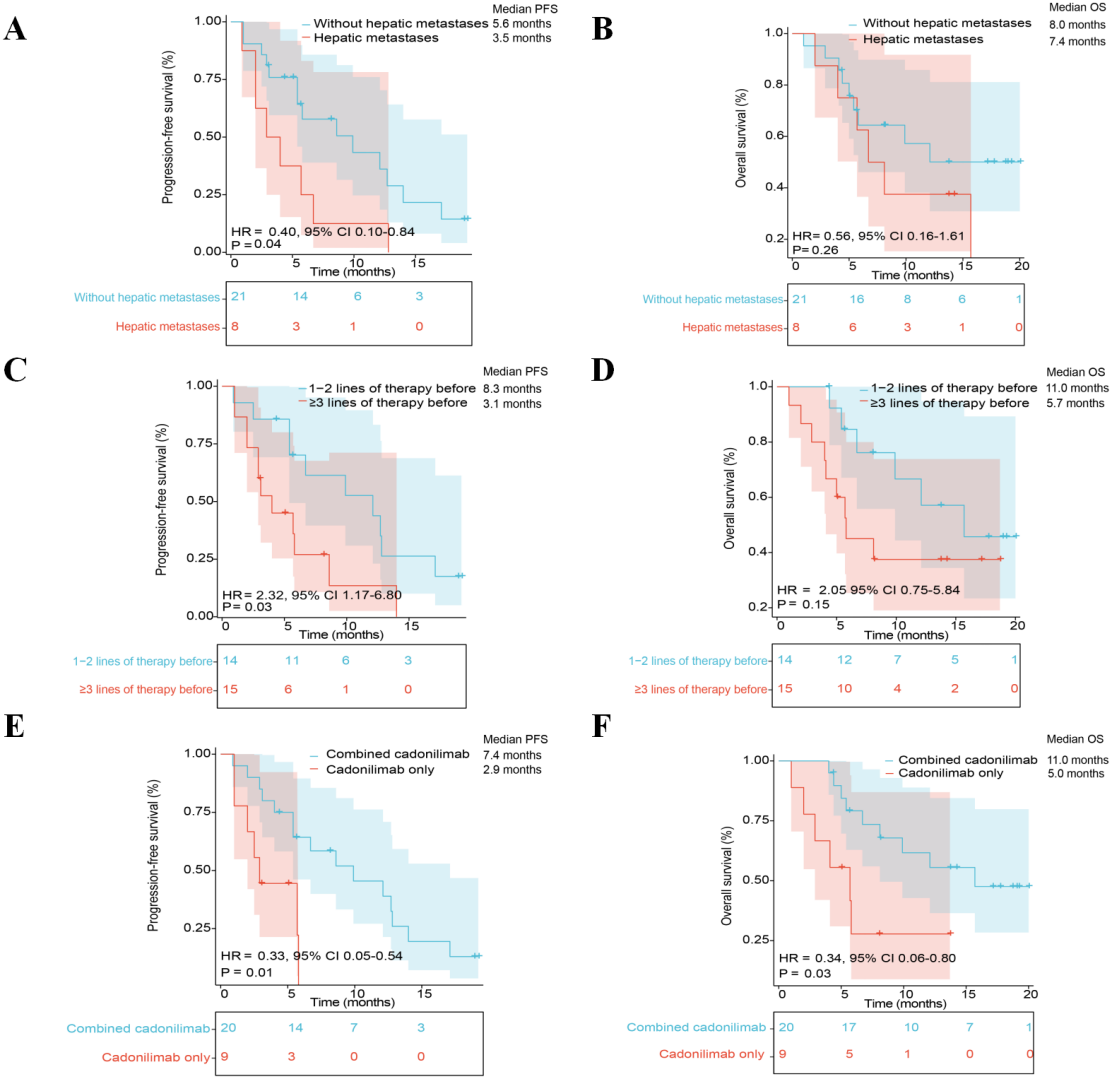
Survival outcomes stratified by clinical characteristics. **(A, B)** Progression-free survival (PFS) and overall survival (OS) comparisons between patients with *versus* without hepatic metastases. **(C, D)** Impact of prior treatment burden on survival: one to two *versus* three or more therapeutic lines. **(E, F)** Therapeutic advantage of combination regimens *versus* cadonilimab monotherapy. All analyses were derived from Kaplan–Meier estimates with log-rank testing. *HR*, hazard ratio.

Subgroup analyses were performed in an exploratory manner to assess whether the clinical outcomes were consistent across key patient characteristics. Patients without hepatic metastases showed a numerically longer PFS compared with those with liver involvement (median = 5.6 *vs*. 3.5 months, HR **=** 0.40, 95%CI **=** 0.10–0.84, *p* = 0.04), while the OS appeared similar between the two groups (8.0 *vs*. 7.4 months, HR **=** 0.56, 95%CI **=** 0.16–1.61, *p* = 0.26) (**Figures 3A, B**). Notably, patients who had received three or more prior treatment lines exhibited a shorter median PFS relative to those treated with one to two lines (3.1 *vs*. 8.3 months, HR **=** 2.32, 95%CI **=** 1.17–6.80, *p* = 0.03), while the median OS showed no clear difference (5.7 *vs*. 11.0 months, HR **=** 2.05, 95%CI **=** 0.75–5.84, *p* = 0.15) (**Figures 3C, D**). Moreover, when examining the treatment modalities during rechallenge, combination therapy showed a trend toward longer PFS (7.4 *vs*. 2.9 months, HR **=** 0.33, 95%CI **=** 0.05–0.54, *p* = 0.01) and OS (11.0 *vs*. 5.0 months, HR **=** 0.34, 95%CI **=** 0.06–0.80, *p* = 0.03) compared with cadonilimab monotherapy (**Figures 3E, F**). However, given the limited sample size and the heterogeneity in concomitant treatments, these differences should be interpreted with caution.

### Adverse events

Treatment-related adverse events (TRAEs) were observed in 24 patients (82.8%), including nine cases (31.0%) of severe (grade ≥3) AEs. The most frequent grade ≥3 toxicities included anemia (8/29, 27.6%), leukopenia (4/29, 13.8%), and neutropenia (4/29, 13.8%). IrAEs occurred in 55.2% (16/29) of the participants. Of note is that the severe irAEs (grade ≥3) were restricted to myocarditis (2/29, 6.9%). Only one patient (3.4%) discontinued treatment due to intolerable AEs (**Table 3**).

**Table 3:** Adverse events in immune rechallenge patients.

Adverse events	Total	Grade 1	Grade 2	Grade 3	Grade 4
Anemia	16 (55.2)	4 (13.8)	4 (13.8)	7 (24.1)	1 (3.4)
Decreased white blood cell count	11 (37.9)	5 (17.2)	2 (6.9)	1 (3.4)	3 (10.3)
Decreased neutrophil count	9 (31.0)	3 (10.3)	2 (6.9)	1 (3.4)	3 (10.3)
Hypokalemia	4 (13.8)	2 (6.9)	1 (3.4)	0 (0)	1 (3.4)
Hypomagnesaemia	6 (20.7)	5 (17.2)	0 (0)	1 (3.4)	0 (0)
Decreased platelet count	6 (20.7)	3 (10.3)	0 (0)	1 (3.4)	2 (6.9)
Immune-related adverse events					
Immune-mediated hypothyroidism	5 (17.2)	2 (6.9)	3 (10.3)	0 (0)	0 (0)
Immune-mediated Rash	2 (6.9)	0 (0)	2 (6.9)	0 (0)	0 (0)
Immune-mediated hypocorticism	3 (10.3)	2 (6.9)	1 (3.4)	0 (0)	0 (0)
Immune-mediated myocarditis	2 (6.9)	0 (0)	0 (0)	2 (6.9)	0 (0)

Values presented are *n* (percentage).

Surprisingly, prior exposure to three or more therapeutic lines did not increase the risk of TRAEs [40.0% (6/15) of grade 2–4 events]. The incidence of grade 3–4 TRAEs was 60.0% in the cadonilimab ± chemotherapy group and was 33.3% in the cadonilimab + anti-angiogenic-therapy group, which were higher compared with the 22.2% observed in the cadonilimab monotherapy group. In addition, individuals with hepatic metastases experienced more grade 2–4 TRAEs compared with the overall cohort (75.0% *vs*. 55.2%) (**Table 4**).

**Table 4:** Safety analysis of the subgroups.

TRAE	Patients, *n* (%)					
	All (*N* = 29)	Hepatic metastases (*n* = 8)	Previous lines of therapy ≥3 (*n* = 15)	Cadonilimab monotherapy (*n* = 9)	Combination with CT (*n* = 10)	Combination with AT (*n* = 18)
Grade 1	8 (27.6)	1 (12.5)	6 (40.0)	4 (44.4)	1 (10.0)	3 (16.7)
Grade 2	7 (24.1)	3 (37.5)	4 (26.7)	1 (11.1)	2 (20.0)	6 (33.3)
Grade 3	6 (20.7)	2 (25.0)	1 (6.7)	0 (0.0)	5 (50.0)	5 (27.8)
Grade 4	3 (10.3)	1 (12.5)	1 (6.7)	2 (22.2)	1 (10.0)	1 (5.6)

TRAEs presented in this table reflected the most severe grade experienced by each patient.

*CT*, chemotherapy; *AT*, anti-angiogenic therapy.

## Discussion

This multi-institutional retrospective study suggests that cadonilimab offers promising survival outcomes and manageable toxicity in ICI-pretreated R/M CC patients, with a median PFS of 5.8 months and OS of 12.1 months. These findings highlight cadonilimab-based regimens as a potential treatment strategy for R/M CC that failed mono-immunotherapy. Importantly, favorable disease control was observed in 3 of the 4 PD-L1-negative patients. Subgroup analysis suggests that liver metastasis, three or more prior treatment lines, and cadonilimab monotherapy may be associated with worse survival outcomes. However, given the small sample size, these findings should be considered exploratory and intended to generate hypotheses for prospective validation in larger studies.

Rechallenge with immunotherapy after initial failure has shown promising clinical benefits (27). Compared with prior second-line studies in CC, such as the phase III trial of cemiplimab monotherapy in R/M CC (ORR = 17.1%) (11) and the AdvanTIG-202 trial of ociperlimab (anti-TIGIT mAb) + tislelizumab combination (ORR = 23.2%) (28), our findings in this later-line rechallenge setting are notable. In this cohort, rechallenge with cadonilimab demonstrated an ORR of 24.1%, which is comparable to the ORR observed with second-line monotherapy in CC. Existing studies on immunotherapy rechallenge in CC are limited. A study in four patients with CC rechallenged with anti-PD-1 monotherapy showed a DCR of only 25% (29), while another study involving seven CC patients treated with PD-1 monotherapy reported one PR and two SD cases (30). In comparison, our study demonstrated the positive impact of dual-targeting strategies, with cadonilimab rechallenge achieving a DCR as high as 55.2%. Similar trends have been observed in melanoma (31), where rechallenge with dual antibodies achieved a DCR of 45% (21/47) (32), and in non-small cell lung cancer (20, 33), where cadonilimab rechallenge in patients who failed immunotherapy led to a median PFS of 1.9 months (95%CI = 1.8–2.0) (21). These findings underscore the significant potential of cadonilimab as a promising rechallenge strategy for R/M CC. However, given the exploratory results, larger randomized controlled trials are needed to validate the efficacy of cadonilimab as an immunotherapy rechallenge strategy for R/M CC.

The underlying mechanisms that explain why dual immunotherapy rechallenge could still be effective in previously ICI-treated patients are not fully clear. The possible explanations are as follows. Firstly, preclinical and clinical studies have shown synergistic antitumor activity with the combination of PD-1 and CTLA-4 blockade (34), demonstrating superior outcomes compared with monotherapy. This superior antitumor activity could broaden its therapeutic application to PD-L1-negative CC populations (23, 35). Our study observed a DCR of 75% (3/4) in patients with CPS <1. However, these results should be considered a preliminary trend observed in an exploratory analysis, which require further validation in larger cohorts. Secondly, dual blockade of CTLA-4 and PD-1 interacts with tumor-infiltrating lymphocytes (TILs) through distinct but complementary mechanisms, which can transform the immunosuppressive TME (36, 37). During T-cell priming, CTLA-4 suppresses the T-cell activation through intrinsic and extrinsic mechanisms, notably by competitively inhibiting CD28–B7 interactions. In contrast, PD-1 primarily functions in peripheral tissues by recruiting tyrosine phosphatases to attenuate T-cell receptor (TCR) signaling following PD-L1/PD-L2 engagement (38). At the cellular level, this functional dichotomy manifests as differential modulation of the T-cell subsets. Anti-CTLA-4 antibodies promote Th1-polarized CD4^+^ effector T cells and deplete regulatory T cells (Tregs), while anti-PD-1 antibodies preferentially rescue exhausted CD8^+^ cytotoxic T cells by reversing the TCR signal inhibition within the TME (39). This multilayered synergy ultimately generates a broader and more durable tumor-specific immunity than that achieved by monotherapy. In addition, combination strategies could potentially shift the tumor immune microenvironment from “cold” to “hot,” thereby enhancing the T-cell infiltration and tumor immunogenicity (40). However, all of the above remain speculative based on current research, and further fundamental studies and clinical trials are warranted to better understand the underlying mechanisms.

Could the safety of dual immunotherapy rechallenge be adequately assured? Current evidence suggests that the incidence of high-grade irAEs does not increase with ICI rechallenge compared with the initial ICI therapy (41). Similarly to our study, the AEs observed in patients were tolerable. Notably, the incidence of grade 3–4 AEs in participants undergoing cadonilimab monotherapy rechallenge was 22.2% (6/19), comparable to that of the 28% reported in the COMPASSION-03 study (16). It is noteworthy that two patients (6.9%) in our cohort developed grade ≥3 immune-mediated myocarditis, which should prompt caution among clinicians when dealing with immune rechallenge patients (27). In the context of multi-line ICI treatment, the exhaustion of Tregs may lead to uncontrolled immune suppression, triggering severe inflammation and cardiac damage. Furthermore, chronic inflammation induced by immunotherapy can result in myocardial fibrosis and cardiac remodeling, representing another form of long-term cardiotoxicity, which may ultimately progress to heart failure or chronic cardiomyopathy (42). Although cadonilimab exhibits encouraging therapeutic efficacy during ICI rechallenge, with manageable toxicity, each case must be evaluated meticulously. A risk–benefit assessment, made through informed discussions between patients and physicians, should serve as the foundation of treatment decisions (43).

### Limitations

Despite the promising results, certain limitations must be recognized. Firstly, in addition to its retrospective, single-country design, the relatively small sample size (*n* = 29) represents a major limitation of this study, potentially introducing selection bias and restricting the generalizability of the findings. Furthermore, due to the small sample size, we were unable to perform propensity score matching or multivariable adjustments to account for the impact of prior treatment lines. Therefore, the subgroup findings should be regarded as exploratory and hypothesis-generating and should be interpreted with caution. Thirdly, all patients were enrolled prior to the inclusion of cadonilimab in the 2024 National Reimbursement Drug List in China. The high pre-reimbursement cost likely influenced the treatment accessibility and patient selection, which may affect the generalizability of our findings. Finally, due to the challenges in a retrospective study, we were unable to conduct exploratory analyses of potential biomarkers, such as the tumor mutational burden (TMB), the microsatellite instability (MSI), or the human papillomavirus (HPV) status.

### Future prospects

Notwithstanding these limitations, our findings provide a rationale for future investigations. The recent inclusion of cadonilimab in the National Reimbursement Drug List in China is a positive step toward improving its affordability. Consequently, it will be more feasible to validate the efficacy and safety of rechallenge with cadonilimab in prospective, multicenter, large-scale clinical studies. Furthermore, understanding the immunological mechanisms underlying cadonilimab rechallenge and identifying its clinical biomarkers should be prioritized in future clinical research. Multiple analyses have shown that a high TMB is associated with better responses to ICIs, including PD-1 and CTLA-4 inhibitors. Patients with MSI-H tumors, which often result in increased TMB, have shown robust antitumor activity (44). Exploratory analyses of potential biomarkers such as TMB, MSI, TILs, and peripheral immune characteristics would further contribute to advancing the field of translational research and help identify patients most likely to benefit from rechallenge strategies.

## Conclusion

The study might open up a new treatment path and hope for CC patients with immunotherapy failure. Immune rechallenge with cadonilimab has improved the survival outcomes, with lower toxicity, making it a promising option for patients with R/M CC. Further investigations are merited to provide more medical evidence and explore its underlying mechanisms.

## Data Availability

The raw data supporting the conclusions of this article will be made available by the authors, without undue reservation.
